# Local and Systemic Regulation of Plant Root System Architecture and Symbiotic Nodulation by a Receptor-Like Kinase

**DOI:** 10.1371/journal.pgen.1004891

**Published:** 2014-12-18

**Authors:** Emeline Huault, Carole Laffont, Jiangqi Wen, Kirankumar S. Mysore, Pascal Ratet, Gérard Duc, Florian Frugier

**Affiliations:** 1 Institut des Sciences du Végétal (ISV), CNRS, UPR2355, Gif-sur-Yvette, France; 2 Plant Biology Division, The Samuel Roberts Noble Foundation, Ardmore, Oklahoma, United States of America; 3 INRA, UMR1347 Agroécologie, Dijon, France; The University of North Carolina at Chapel Hill, United States of America

## Abstract

In plants, root system architecture is determined by the activity of root apical meristems, which control the root growth rate, and by the formation of lateral roots. In legumes, an additional root lateral organ can develop: the symbiotic nitrogen-fixing nodule. We identified in *Medicago truncatula* ten allelic mutants showing a *compact root architecture* phenotype (*cra2*) independent of any major shoot phenotype, and that consisted of shorter roots, an increased number of lateral roots, and a reduced number of nodules. The *CRA2* gene encodes a Leucine-Rich Repeat Receptor-Like Kinase (LRR-RLK) that primarily negatively regulates lateral root formation and positively regulates symbiotic nodulation. Grafting experiments revealed that CRA2 acts through different pathways to regulate these lateral organs originating from the roots, locally controlling the lateral root development and nodule formation systemically from the shoots. The CRA2 LRR-RLK therefore integrates short- and long-distance regulations to control root system architecture under non-symbiotic and symbiotic conditions.

## Author Summary

Despite the essential functions of roots in plant access to water and nutrients, root system architecture has not been directly considered for crop breeding improvement, but it is now considered key for a “second green revolution.” In this study, we aimed to decipher integrated molecular mechanisms coordinating lateral organ development in legume roots: lateral roots and nitrogen-fixing symbiotic nodules. The *compact root architecture 2* (*cra2*) mutant form an increased number of lateral roots and a reduced number of symbiotic nitrogen-fixing nodules. This mutant is affected in a CLAVATA1-like Leucine-Rich Repeat Receptor-Like Kinase (LRR-RLK) that has not previously been linked to root development. Grafting experiments showed that CRA2 negatively controls lateral root formation and positively controls nodule development through local and systemic pathways, respectively. Overall, our results can be integrated in the framework of regulatory pathways controlling the symbiotic nodule number, the so-called “Autoregulation of Nodulation” (AON), involving another LRR-RLK that also acts systemically from the shoots, SUNN (Super Numeric Nodules). A coordinated function of the CRA2 and SUNN LRR-RLKs may thereby permit the dynamic fine tuning of the nodule number depending on the environmental conditions.

## Introduction

Plant growth requires the continuous development of the root system and its adaptation to changing environmental soil conditions. Mechanisms controlling root system architecture at the whole-plant level, including the systemic coordination of shoot and root development, are key breeding targets for maintaining crop productivity under adverse stress conditions but remain poorly understood [1]. Root system architecture is a consequence of the sustained activity of root apical meristems, leading to indeterminate root growth as well as the *de novo* formation of lateral organs. In legume (*Fabaceae*) plants, the root system can form two types of lateral organs depending on the environmental conditions: lateral roots and symbiotic nitrogen-fixing nodules [2]–[4]. Lateral root initiation, emergence and growth depend on water and nutrient availability and are regulated by a combination of local and systemic pathways [5]. Symbiotic nodules are formed under nitrogen-deprived conditions when the specific *Rhizobium* spp. soil bacteria are present in the rhizosphere [6]–[7]. In both types of lateral organogeneses, cell divisions are activated in specific tissues (pericycle, endodermis and cortex) above the growing root tip [2]–[4], [8]. Root tissues contributing to primordium formation are, however, different depending on the plants and organs: in the *Medicago truncatula* model legume, lateral root primordia mainly develop from pericycle cell divisions, whereas the nodule primordia that are induced by *Sinorhizobium meliloti* mainly derive from the inner cortex. Both types of primordia will then subsequently emerge from the parental root and establish a meristematic stem cell niche ensuring their indeterminate growth.

To control meristematic activity, cell differentiation, and lateral organ initiation, non-cell autonomous cues are essential to carry positional information, which can be informed either by mobile phytohormones, small RNAs or peptides [9]–[11]. Among peptides, several CLAVATA 3/EMBRYO-SURROUNDING REGION (like) peptides (so called CLE peptides; [10]) that are perceived by Leucine-Rich Repeats – Receptor-Like Kinases (LRR-RLKs) are involved in local and long-distance (systemic) pathways controlling the development of different plant organs. First, several CLE peptide/LRR-RLK receptor modules carry positional information across a few cell layers to control the cell fate in different *Arabidopsis thaliana* meristems. The founding example is the CLAVATA3 (CLV3) peptide, which is perceived by the CLV1 receptor to control the shoot apical meristem stem cell niche [12], [13] as well as columella cell differentiation in the Root Apical Meristem (RAM; [14]–[16]). A second example is the Tracheary element Differentiation Inhibitory Factor (TDIF) peptide, which is perceived by the Phloem Intercalated with Xylem (PXY) receptor, controlling stem cell proliferation/differentiation transition in the cambium meristem and therefore vasculature differentiation and organ thickening [17]–[19].

In legume plants, an additional CLE/LRR-RLK module controlling root lateral organs number was identified through grafting experiments as performing a long-distance systemic function from the shoots [7]. Mutants affecting orthologous LRR-RLKs that are closely related to CLV1 in Arabidopsis (SUNN, Super Numeric Nodules in *M. truncatula*; NARK, Nodule Autoregulation receptor Kinase in soybean; and HAR1, Hypernodulation and Aberrant Root 1 in *Lotus japonicus*; [20]–[23]) form an increased number of symbiotic nitrogen-fixing nodules depending on the receptor function in the shoots. In addition, the Lotus KLAVIER (KLV) LRR-RLK, which is closely related to the Arabidopsis RECEPTOR-LIKE PROTEIN KINASE 2 (RPK2)/TOADSTOOL 2 (TOAD2) that is functionally linked to anther and embryo development, is involved in the same systemic AON pathway as is HAR1 [24]. In *M. truncatula* and *L. japonicus*, CLE peptides that are specifically produced in nodulated roots can negatively regulate the nodule number depending on these LRR-RLK receptors [25]–[27]. This CLE peptide/LRR-RLK module therefore participates in the systemic “Autoregulation of Nodulation” (AON; [22]) pathway, which may involve a direct root-to-shoot CLE peptide transport and receptor binding in the shoots, as recently proposed [28]. Interestingly, an increased number of emerged lateral roots was reported in the Lotus *har1* mutant under both symbiotic and non-symbiotic conditions [29]. Similarly, KLV also has a non-symbiotic function in the local regulation of SAM maintenance [24]. Collectively, these results indicate that CLE peptide/LRR-RLK signaling modules regulate the development of various organs using either local and/or systemic pathways.

## Results

### The *COMPACT ROOT ARCHITECTURE 2* (*CRA2*) gene negatively regulates lateral root development

**Figure 1:**
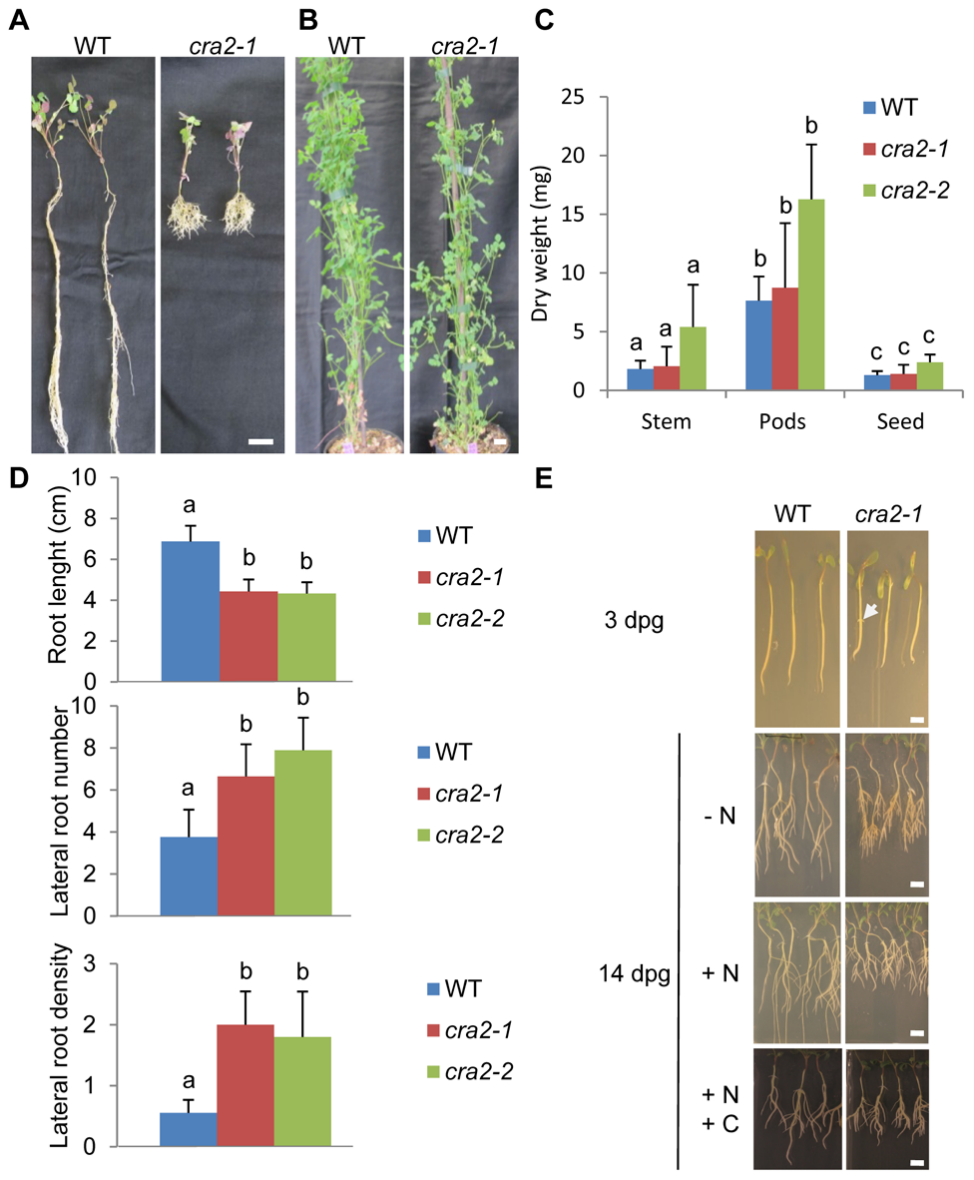
The “*compact root architecture 2*” (*cra2*) mutants have short roots and more lateral roots independently of the growth conditions. **A** and **B**, Representative examples of wild-type (WT) and *cra2-1* plants that were grown in the greenhouse for one month on a perlite-sand mixture (**A**) or for three months on soil (**B**). Bar  = 1 cm in A, 10 cm in B. **C**, Quantification of the stems, pods and seeds dry weight of the WT, *cra2-1* and *cra2-2* plants that are shown in (**B**). The error bars represent confidence intervals (α = 5%). A Kruskal and Wallis test was used to determine the significant differences (indicated by letters, α<5%; n = 10). **D**, Quantification of the root length (upper graph), lateral root number (middle graph) and lateral root density (lower graph) of the WT, *cra2-1* and *cra2-2* plants that were grown *in vitro* for 10 days post-germination (dpg) on an N-deprived “i” medium [42]. The error bars represent confidence intervals (α = 1%). A Kruskal and Wallis test was used to determine the significant differences (indicated by letters, α<1%; n>25). **E**, Representative examples of the WT and *cra2-1* plants that were grown *in vitro* for three dpg on an N-deprived “i” medium [42] or for 14 dpg on the same medium (- N), on an N-rich medium (+N, Fahraeus with NH_4_NO_3_ 10 mM; [43]), or on an N- and C-rich medium (+N+C, “Lateral Root Inducing Medium; [42]). Note that in *cra2*, the lateral roots were already emerged at three dpg (arrowhead) and that the “compact root system architecture” phenotype was detectable independently of the growth medium. Bars  = 0,5 cm.

**Figure 2:**
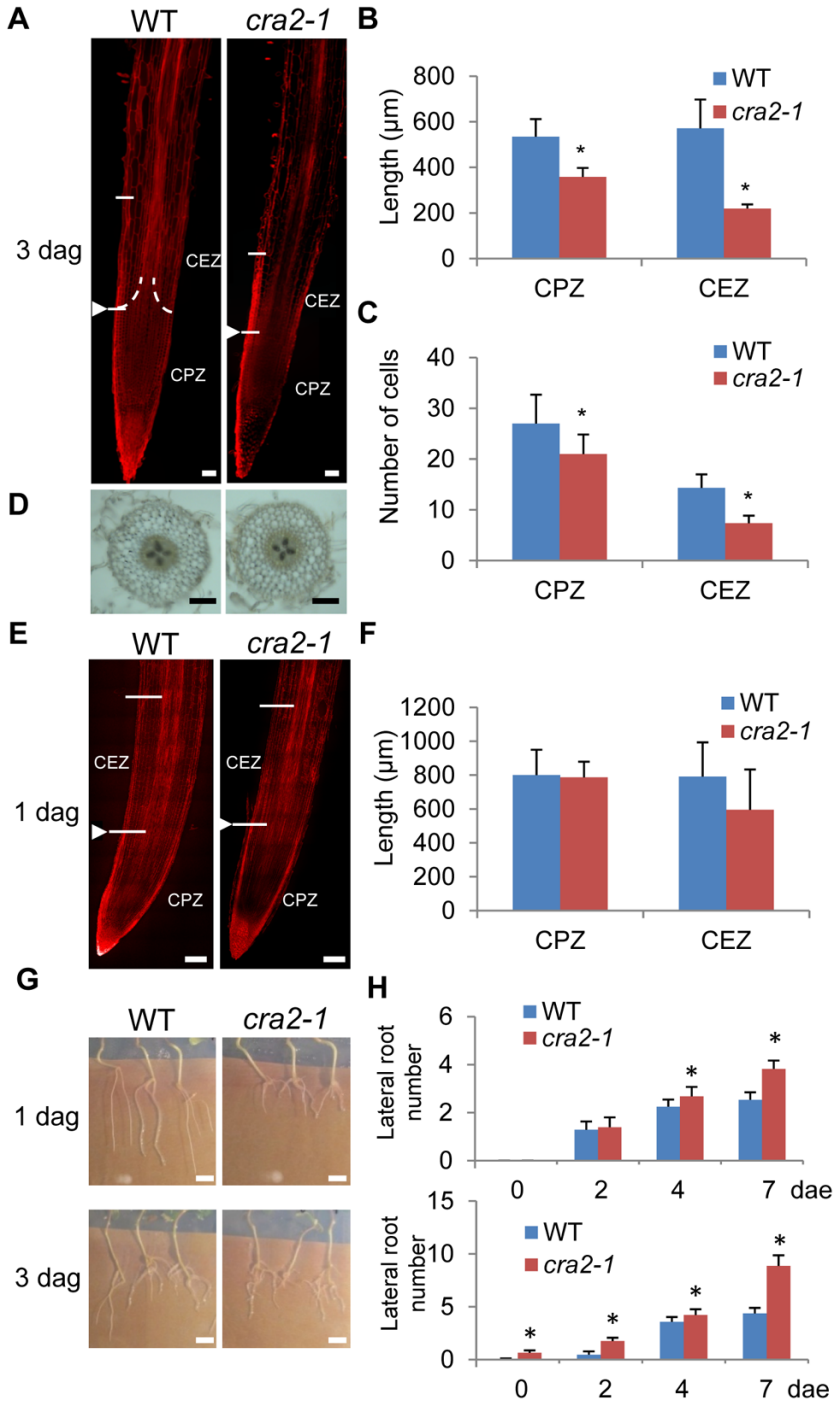
The *cra2* lateral root and root apical meristem phenotypes can be disconnected. **A** and **E**, Representative examples of the wild-type (WT) and *cra2-1* Root Apical Meristems (RAM, stained with Propidium Iodide to visualize cell walls) three days post germination (dpg; **A**) or one dpg (**E**). The arrowhead indicates the apical position of the “cone-shaped” transition zone (as defined in S2 Fig.) between the cell proliferation zone (CPZ) and the cell elongation zone (CEZ). Bar  = 100 µm. **B, F**, Quantification in the WT and *cra2-1* roots of CPZ and CEZ length at three dpg (**B**) or one dpg (**F**). **C**, Quantification in the WT and *cra2-1* roots of the cells number in the same zones at three dpg. In **B**, **C**, and **F**, the error bars represent standard deviation, and a Mann-Whitney test was used to determine the significant differences between genotypes (*, α<5%, n = 10). **D**, Transversal sections of three dpg WT and *cra2-1* roots showing a similar radial organization of cell layers. Bar  = 100 µm. **G**, Representative examples of the WT and *cra2-1* root system architecture seven days post excision (dpe) of the RAM. The excision was performed either one day after germination (dpg, upper pictures) or three dpg (lower pictures). Bar  = 1 cm. **H**, Quantification of the lateral roots at two, four and seven days post excision (dpe) of the RAM in WT or *cra2-1* plants. As shown in (**G**), the meristems were excised at either one dpg (upper graph) or three dpg (lower graph). The error bars represent the confidence interval (α = 5%), and a Mann-Whitney test was used to determine significant differences between the genotypes for each time point (*, α<5%; n>25).

Forward genetic screens that were performed on a *M. truncatula Tnt1* insertional mutant collection [30]–[31] identified seven mutant lines with a wild-type shoot development and a “compact root system architecture” (so called “*cra*”; Fig. 1A–C and S1A Fig.). Segregation analyses of this root phenotype revealed a 3∶1 WT:mutant ratio (chi2 test, p<0.05, n = 30), suggestive of a single locus recessive mutation. Allelism tests (S1B Fig.) indicated that all of the mutants were affected in the same locus but differed from the previously identified *cra1* mutant showing partially similar phenotypes [32] and were therefore named *cra2*. Detailed quantitative *in vitro* analyses revealed that the *cra2* phenotype consists, compared to the WT, of shorter roots with an increased number of emerged lateral roots (Fig. 1D–E). Accordingly, lateral roots were observed three days post germination (dpg; Fig. 1E) in contrast to WT plants. This root phenotype was observed independently of the growth and nutrient conditions that were used (greenhouse versus *in vitro*, Fig. 1A, E; with or without nitrogen or carbon sources, Fig. 1E). In addition to the faster emergence of lateral roots, we observed a reduction in the primary root growth, which prompted us to analyze the structure of the RAM (Fig. 2A–C). In contrast to the *A. thaliana* model, *M. truncatula* roots have an open meristem, and the transition between cell proliferation and differentiation is more progressive. In addition, cells from diverse files elongate at a slightly different distance from the root apex, leading to a cone-shaped transition zone (Fig. 2A, detail of the transition zone in S2 Fig.). When *cra2* RAMs were observed three dpg, both the cell proliferation and elongation zones were reduced, and a lower number of cells was observed in the two zones (Fig. 2 A–C). Root patterning, however, evaluated based on both longitudinal and transversal sections, seemed unaffected (Fig. 2A–B and D). In addition, amyloplast accumulation in differentiated root cap cells and the expression of the RAM stem cell niche marker WOX5 (WUSCHEL-related homeobox 5; [33]–[35]) were both detected in *cra2* (S3 Fig.), suggesting the maintenance of RAM cell identity. Among several hypotheses that could explain the *cra2* root system architecture phenotype, we tested whether a RAM activity defect could indirectly lead to increased branching as compensation or if the reduced meristematic activity could be a consequence of the enhanced formation of lateral roots. An analysis of root apices at one dpg, i.e., before any lateral root primordium could be detected in the *cra2* mutant, revealed no significant difference in the size of the cell proliferation and elongation zones between WT and mutant roots (Fig. 2E, F). Compared to previous observations of roots at three dpg (Fig. 2A, B), this result indicates that a RAM defect does not precede the occurrence of the lateral root phenotype. As an independent approach, we experimentally removed the RAM at one or three dpg and followed the kinetics of lateral root formation (Fig. 2G, H). The *cra2* mutant showed an increased ability to form lateral roots whether RAM excision occurred before or after lateral root initiation. Collectively, these results suggest that the *cra2* RAM phenotype can be disconnected from its enhanced ability to form lateral roots.

### The *CRA2* gene positively regulates symbiotic nodule development

**Figure 3:**
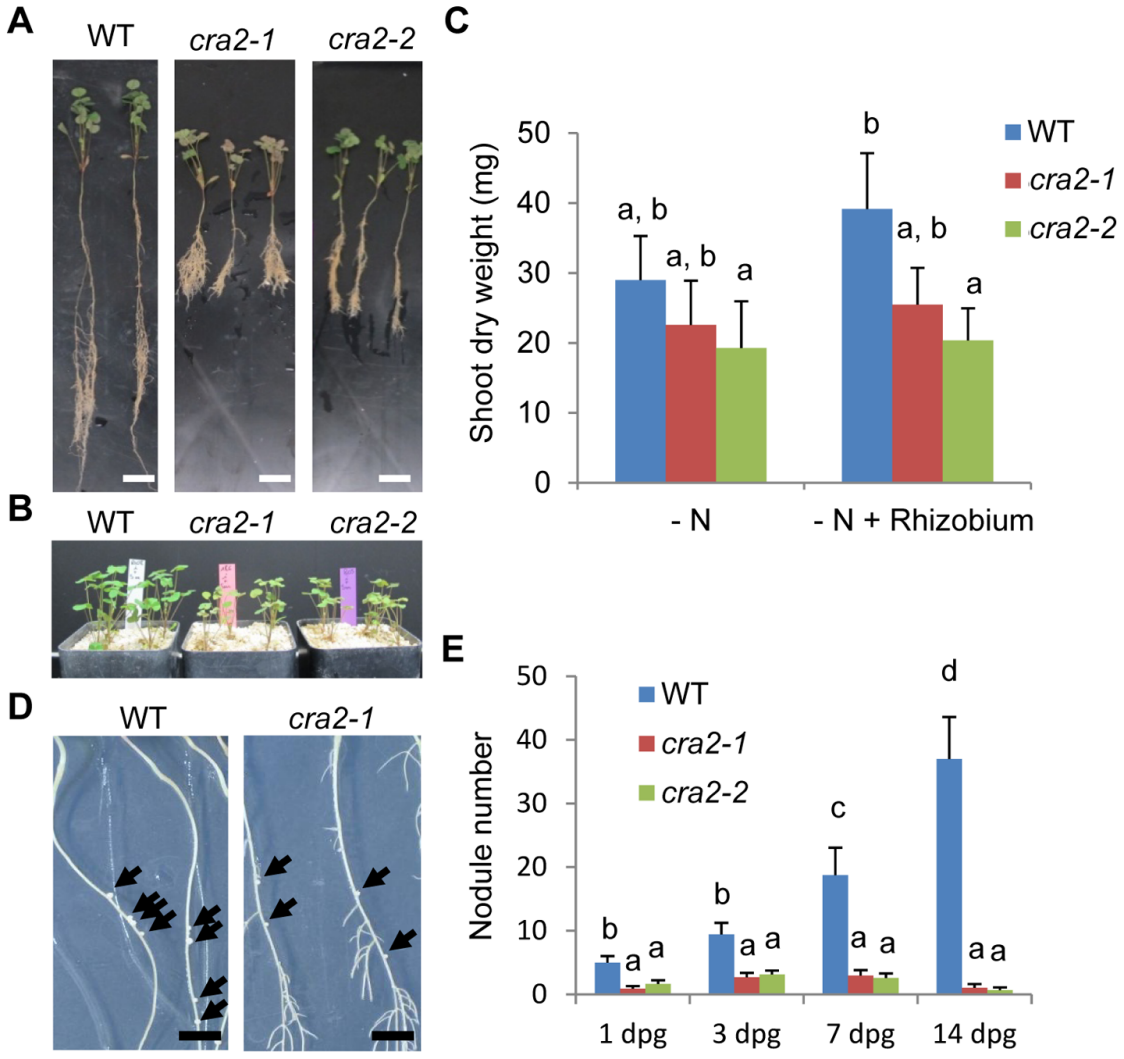
The *cra2* mutants have reduced symbiotic nodulation. **A**, Representative examples of the wild-type (WT), *cra2-1* and *cra2-2* plants that were grown in the greenhouse on a perlite-sand mixture for two weeks (**A**) or one month (**B**) on an N-deprived medium (“i”, [42]) and then inoculated by *Sinorhizobium meliloti* 1021 for 14 days. Bar  = 1 cm. **C**, Quantification of the shoot dry weights of the WT, *cra2-1* and *cra2-2* plants that are shown in (**A**). The error bars represent confidence intervals (α = 5%). A Kruskal and Wallis test was used to determine the significant differences (indicated by letters, α<5%; n = 30). **D**, Representative examples of the WT and *cra2-1* plants that were grown for one week *in vitro* on an N-deprived medium (“i”, [42]) and then inoculated by *S. meliloti* 1021 for 14 days. The arrowheads indicate the position of the nodules. Bar  = 1 cm. **E**, Quantification of the nodule number 14 days post inoculation with *S. meliloti* in the roots of the WT and *cra2-1* plants that were grown *in vitro*. The inoculation was performed between one and 14 days post germination (dpg). The error bars represent confidence intervals (α = 5%). Significant differences were determined using a Mann-Whitney test (α<5%; n>20).

In addition to root branching, legume roots can adapt to environmental conditions by developing another root lateral organ, the nitrogen-fixing symbiotic nodule. An analysis of *cra2* roots under symbiotic conditions revealed that a similar “compact root architecture” phenotype was observed (Fig. 3A) and that mutant shoot growth was similar with or without *Rhizobium* inoculation (Fig. 3B, C). *cra2* plants, however, developed a strongly reduced number of symbiotic nodules (Fig. 3 D, E). This low nodulation phenotype could be either linked to a direct CRA2 function in regulating nodulation or may reflect that the strongly altered “compact root architecture” phenotype (e.g., Fig. 3A) indirectly hampers nodule formation. Using a symbiotic infection kinetic analysis (1 to 14 dpg), we showed that *Rhizobium* inoculation as early as 1 or 3 dpg led to reduced nodulation (Fig. 3E), indicating that the *cra2* nodulation phenotype was independent of the strength of the lateral root phenotype. The few symbiotic nodules that formed on *cra2* roots were elongated (S4A Fig.), revealing that a functional nodule meristem was formed. In addition, an analysis of bacterial nitrogenase activity using an Acetylene Reduction Assay (ARA) showed that despite *cra2* plants have a strongly reduced ability to fix atmospheric nitrogen due to their lower number of symbiotic organs (S4B Fig.), *cra2* nodules have a similar specific ARA activity compared to that of WT nodules (S4C Fig.). Overall, the detailed analysis of the mutant phenotypes indicates that, independently of a potential indirect effect of the increased lateral root formation on nodule formation, CRA2 antagonistically regulates lateral root and nodule formation.

### CRA2 regulates root system architecture depending on systemic and local pathways

**Figure 4:**
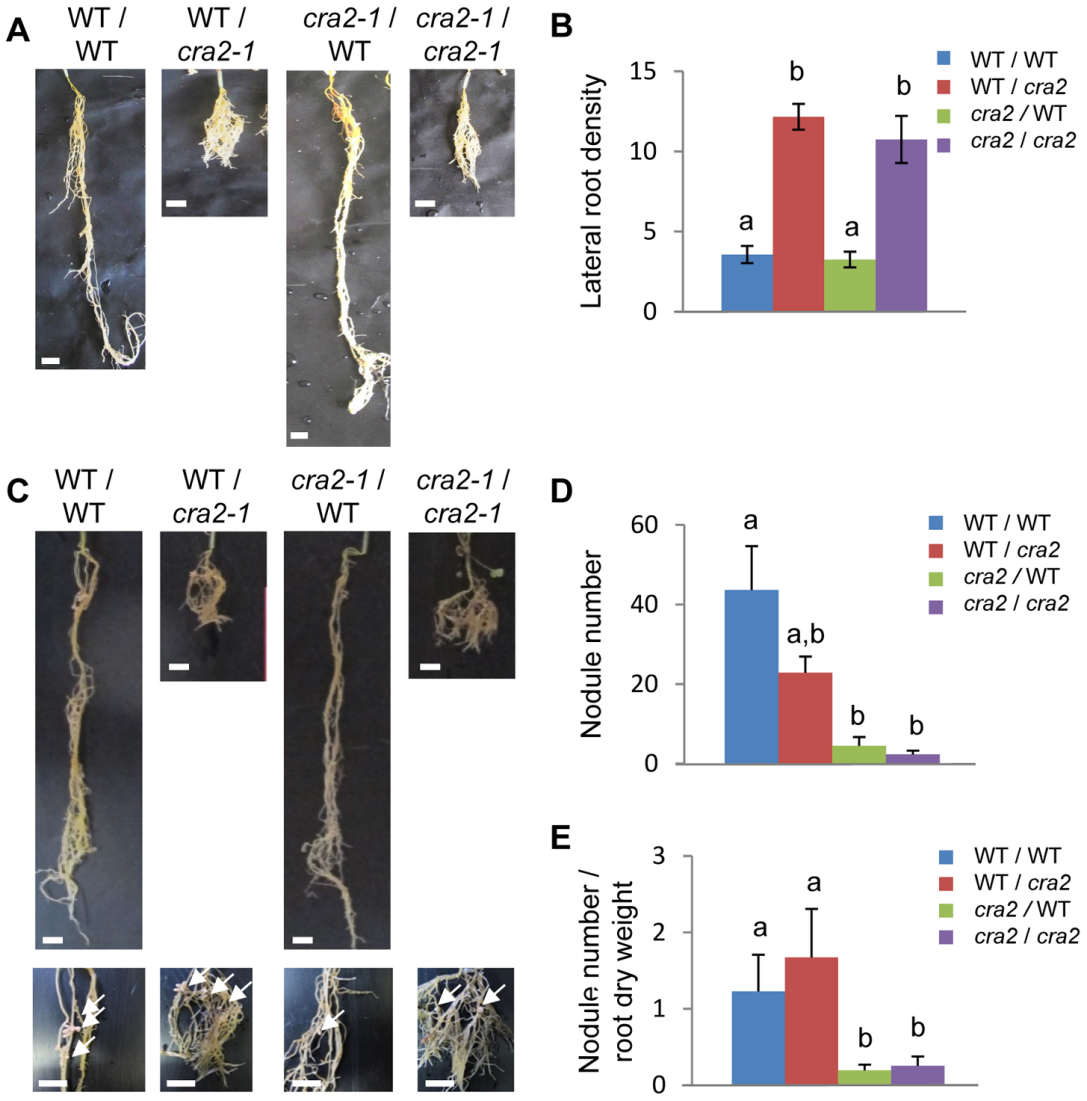
*CRA2* locally regulates lateral root formation and systemically regulates symbiotic nodule formation from the shoots. **A** and **C**, Representative images of different grafting combinations between the wild-type (WT) and *cra2-1* plants that were grown in greenhouse with a perlite-sand mixture for eight weeks on an N-rich medium (**A**) or on an N-deprived medium with *Sinorhizobium meliloti* 1021 (**C**). The shoot/root grafting combinations are indicated above each picture. In panel (**C**), detailed pictures showing nodules (arrows) are included below. Bar  = 1 cm. **B**, Quantification of the lateral root density (number of emerged lateral roots/centimeter of parental root) in the different grafting combinations that are shown in (**A**). **D** and **E**, Quantification of the nodule numbers (**D**) and the nodule number related to the root dry weight (**E**) in the different grafting combinations that are shown in (**C**). In **B**, **D** and **E**, the error bars represent confidence intervals (α = 5%), and the significant differences were determined using a Kruskal and Wallis test (indicated by letters, α<5%; n>10).

As root system architecture is controlled by both systemic and local pathways, we next determined using graftings experiments whether the *cra2* phenotypes depend on roots and/or shoots. Under non-symbiotic conditions, the “compact root architecture” phenotype was recovered specifically when the graft combination included *cra2* mutant roots (Fig. 4A–B) but was independent of the shoot genotype, indicating that *CRA2* expression in roots is sufficient to negatively regulate lateral root formation. Under symbiotic conditions, we surprisingly observed a disconnect between the lateral root and nodulation phenotypes (Fig. 4C–D). Indeed, similar to non-symbiotic conditions, the increased density of the lateral roots was associated with *cra2* mutant roots, but the low nodulation phenotype was observed only in grafting combinations that included *cra2* mutant shoots and was independent of the root genotype. This result indicates that the systemic activity of CRA2 in the shoots positively regulates symbiotic nodule formation in the roots. Strikingly, the *cra2*/WT grafts had a WT root system architecture but developed more than 10 times fewer nodules (Fig. 4C–D). In addition, when the nodule numbers were related to the root dry weight to counterbalance the strong differences existing between WT/WT and WT/*cra2* root systems, the nodulation efficiency became strictly equivalent between these two grafting combinations (Fig. 4E). These results therefore unambiguously demonstrate that the low-nodulation phenotype is not an indirect consequence of an inhibitory signal that is produced in the numerous lateral roots in *cra2*. In addition, this result indicates that the root-dependent regulation of the lateral root number is independent of the shoot-regulation of the nodule number.

### The *CRA2* gene encodes a Leucine-Rich Repeat – Receptor-Like Kinase that has not previously been linked to root or symbiotic nodule development

**Figure 5:**
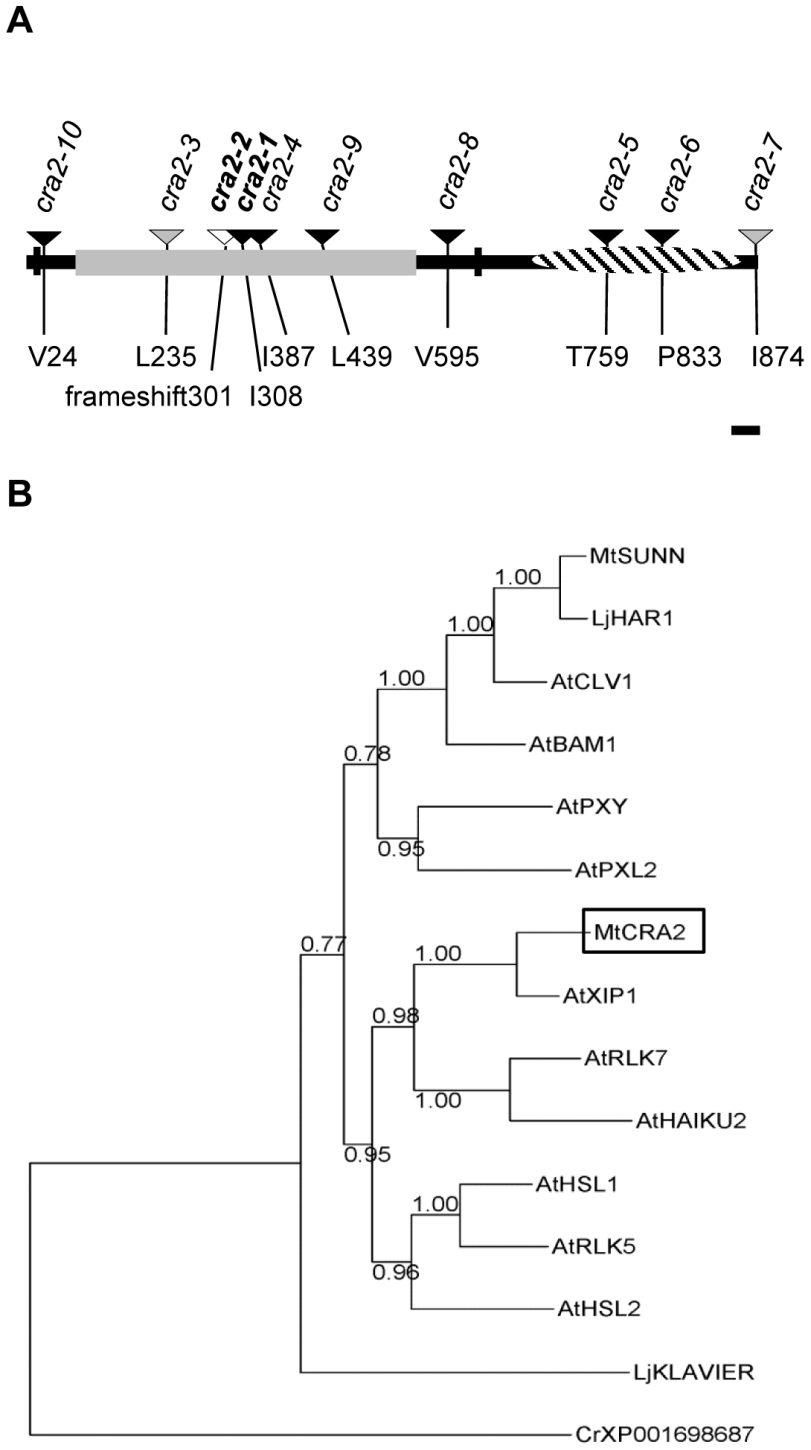
The *CRA2* gene encodes a Leucine-Rich Repeat Receptor-Like Kinase (LRR-RLK). **A**, Structure of the CRA2 protein indicating the 10 mutant alleles (arrowheads) that were identified by forward and reverse genetic screens (the indicated position is related to the predicted ATG) and functional domains. The vertical black bars indicate the predicted transmembrane domains; in grey are the Leucine-Rich Repeats; and the hatched region represents the kinase domain. The black arrowheads represent alleles that are linked to a *Tnt1* retro-element insertion; the grey arrowheads represent another insertional element; and the white arrowhead represents a nucleotide deletion causing a translational frameshift. Bar  = 50 residues. **B**, Phylogenetic tree of selected LRR-RLKs that are related to CRA2 from *Arabidopsis* (subfamily XI) or are functionally characterized in legumes. The sequences were aligned using Muscle, and the regions that were conserved between all of the sequences were defined with Gblocks. The phylogenetic relationships were determined using a maximum likelihood analysis (PhyML), and statistical support for each node was estimated by approximate likelihood ratio tests. The *Chlamydomonas reinhardtii* XP001698687 protein was used to root the tree.

**Figure 6:**
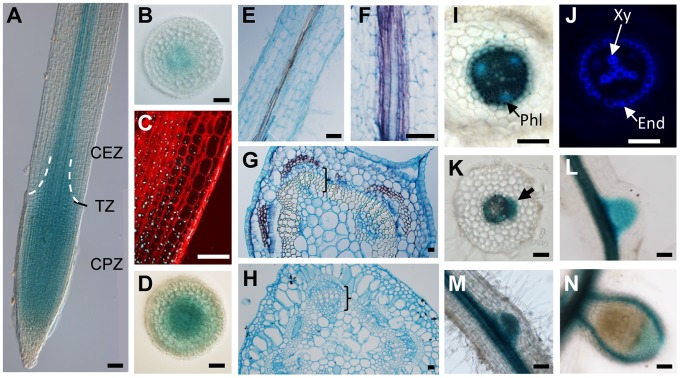
*CRA2* expression in the shoot, root and symbiotic nodules. The spatial expression pattern of *CRA2* was analyzed using a promoter (1,8 kb)-GUS fusion (**A–D** and **I–N**) or by *in situ* hybridization (**E–H**). **A**, A root apex observed in bright field with dichroic illumination (Nomarski). The dotted lines indicate the position of the “cone-shaped” transition zone (TZ) between the cell proliferation zone (CPZ) and the cell elongation zone (CEZ). **B** and **D**, Root transversal sections in the CEZ (**B**) and the CPZ (**D**). **C**, Detail of the root meristem transition zone using a z-stack projection in confocal sections. The cell walls are visualized with a Propidium Iodide counterstaining, and GUS staining appears in reflectance as blue dots. **E–F**, *In situ* hybridization of the *CRA2* transcripts in root longitudinal sections. (**F**) Detail of the purple signal associated with vasculature strands. **G–H**, *In situ* hybridization of the *CRA2* transcripts in stem transversal sections (**G**, purple signal) or with a sense probe used as a negative control (**H**). Brackets indicate vascular bundles. **I–J**, Detail of the stele in the root differentiated region in bright field (**I**) to visualize the GUS staining (in dark blue) and phloem vascular bundles poles (Phl, in turquoise blue) or under UV illumination (**J**, same section as I) to visualize the blue autofluorescence of the xylem vascular bundle poles (Xy) and endodermis (End). **K–L**, Lateral root primordium initiation (**K**, arrow) and emergence (**L**) observed in bright field. **K** is a root transversal section. **M–N**, Nodule primordium (**M**, three days post-inoculation [dpi] with *S. meliloti* 1021) and mature nitrogen-fixing nodule (**N**, 14 dpi) observed in bright field. Bars  = 100 µm.

To identify the gene that is affected in *cra2* mutants, *Tnt1* Flanking Sequence Tags (FSTs) were generated in the different alleles (*cra2-1* to *cra2-7*) to detect *Tnt1* insertions affecting a common genomic sequence in several of these mutant lines (*cra2-1*, *cra2-4* to *cra2-6*; Fig. 5A and S5A–B Fig.). Interestingly, the three other alleles also showed genetic lesions in the same locus, consisting, respectively, in the presence of other *M. truncatula* endogenous insertional elements for *cra2-3* and *cra2-7* and in a point mutation for *cra2-2* leading to a frameshift (S5C-E Fig.). Based on the FSTs that were available from the Noble foundation (http://bioinfo4.noble.org/mutant/), we could identify three additional lines with a *Tnt1* insertion in the same open reading frame (*cra2-8* to *cra2-10*) that showed a “compact root architecture” phenotype (Fig. 5A and S1A Fig.), further confirming that mutation at this locus is causal for the phenotype. The genomic region that was affected in the 10 available *cra2* alleles encodes an LRR-RLK belonging to subfamily XI (Fig. 5B), which, in agreement with the results of the grafting experiment, is expressed in the shoots, roots and nodules (S6 Fig.). Interestingly, this LRR-RLK subfamily contains several other receptors that were identified as regulating plant development *via* local or systemic regulation [10], [36]. An analysis of CRA2 homology with other LRR-RLKs that have been functionally characterized in legumes showed that CRA2 was not closely related to SUNN/HAR1 or KLV and was most closely related to *A. thaliana* XIP1 (Xylem Intermixed in Phloem 1; [37]; Fig. 5B). An Arabidopsis *xip1* mutant was recently described as showing defects in vasculature patterning specifically in the shoots but not in the roots, and no root developmental phenotype was reported [37]. To determine whether XIP1 and CRA2 may nevertheless have related functions, we analyzed the vascular patterning in *cra2* mutant roots and shoots. No significant defect could be detected in the shoot or root cambium structure; in the vascular bundle differentiation, such as the misspecification of xylem and phloem bundles; or in the root or stele diameter (S7 Fig.). These results suggest that XIP1 and CRA2 are not functional homologs. In addition, these results indicate that the *cra2* root and nodulation phenotypes (Fig. 1, 2) are not linked to any detectable defect in vascular bundle patterning. We then analyzed the *CRA2* spatial expression pattern under non-symbiotic and symbiotic conditions using either a transcriptional fusion between an ∼2 kb *CRA2* promoter region and the GUS reporter (Fig. 6A–D and I–N) or *in situ* hybridization (Fig. 6 E–H). Both approaches revealed an expression that was associated with the root stele and vascular bundles (Fig. 6A–B and E–F). Similarly, *CRA2* expression was associated with vascular bundles in the shoots (Fig. 6G–H). This result agrees with the expression pattern of other LRR-RLKs regulating lateral root and nodule numbers in different legumes (HAR1/NARK/SUNN or KLV; 24, [38]–[40]). CRA2 was additionally expressed in the Cell Proliferation Zone (CPZ) of the open RAM (Fig. 6A and C–D). In addition, CRA2 was expressed in the lateral root initiation site as soon as the pericycle divisions could be identified (Fig. 6I–J) and later in whole lateral root primordia during the initiation and emergence stages (Fig. 6K–L). Under symbiotic nodulation conditions, *CRA2* expression was also detected in nodule primordia (Fig. 6M) as well as in mature nodules in relation to peripheral vascular bundles and the apical meristem (Fig. 6N).

## Discussion

CRA2 functions in the regulation of legume root system architecture suggest that this LRR-RLK acts positively in the Autoregulation of Nodulation (AON) pathway. In the current model, the systemic SUNN-dependent AON pathway represses the number of nodules that are formed on the roots depending on the shoot metabolic status, e.g., the amount of carbon skeletons that are provided through photosynthesis and that are required for the subsequent assimilation of fixed nitrogen in the nodules. The SUNN pathway therefore limits the formation of extra nodules depending on environmental cues. When initiated, however, a negative feedback mechanism would be necessary to reset this negative regulation and further permit new symbiotic infection events. The CRA2 LRR-RLK may then participate in the systemic dynamic fine tuning of nodule formation. As both CRA2 and SUNN LRR-RLKs are expressed in the shoot vasculature, it remains to be determined whether and how these proteins act together or independently. As LRR-RLKs have been proposed to form large complexes comprising different members of the family [36] or even other RLKs (e.g., CLV1 with ACR4 in the RAM; [16]), CRA2 and SUNN may interact within a single complex.

The Lotus HAR1-dependent AON pathway was additionally shown to control negatively lateral root formation [20]–[29]. In the *sunn* Medicago mutant, however, the root length is reduced, but no specific function in the regulation of lateral root number has been identified [23]. CRA2 therefore negatively affects lateral roots similarly to HAR1 in Lotus but dissimilarly to SUNN in *M. truncatula*. Interestingly, a shorter root length and a decreased number of lateral roots occurred in the Lotus *klv* mutant, whereas additive effects of *klv* and *har1* were identified for nodulation, indicating their related function in a single AON pathway [40]. In addition, both *har1* and *klv* antagonistic lateral root phenotypes were observed under non-symbiotic conditions [29], [40], similar to the phenotype of CRA2 in *M. truncatula*. This result indicates that these peptide/LRR-RLK regulatory modules acting under non-symbiotic conditions may also control lateral root formation in non-nodulating (non-legume) plants. In Arabidopsis, the ACR4 CRINKLY-RLK regulates lateral root initiation [41], but no LRR-RLK has been yet associated with this developmental process. More generally, despite the close relationships between HAR1, SUNN and CLV1, their mutant phenotypes are not conserved between plant species: the *sunn* and *har1* mutants have divergent lateral root phenotypes, whereas no shoot fasciation phenotype was detected in the *har1* or *sunn* mutant in contrast to *clv1* in *Arabidopsis*. Similarly, despite CRA2 and XIP1 having closely related sequences, no vasculature patterning phenotype was identified in *cra2* compared to *xip1*, and no altered root system architecture phenotype was reported in *xip1*. This result suggests that various sets of LRR-RLKs differentially regulate the ability of root systems to form lateral roots depending on species, even inside the legume family. Alternatively and non-exclusively, different patterns of LRR-RLK gene duplication may have occurred in the different plant genomes, generating functionally divergent or redundant pathways. These scenarios may explain the apparent phenotypic diversification that is observed.

Overall, this study demonstrates that a single LRR-RLK acts locally in roots and systemically in shoots to control root lateral organ development, thereby coordinating at the whole-plant level the plastic development of the root system depending on the changing environmental conditions. To elucidate the opposite effects of the CRA2 pathway on lateral root and nodule formation, the identification of downstream targets will be essential. Among other candidate pathways, cytokinins were previously reported to antagonistically control lateral root and symbiotic nodule formation [42]. Data on crosstalk between signaling peptides and hormones is just emerging, mainly with auxin and cytokinins [10]. Therefore, a remaining challenge will be to integrate the different peptide/receptor modules that are known to regulate lateral root and nodule formation, including the CRA2 pathway, into the framework of hormonal regulation.

## Materials and Methods

### Plant and bacteria material

The *Medicago truncatula* (Gaertn.) plants used in this study were of the R108-4 genotype. The *Tnt1* insertional mutants were generated and screened at the Noble Foundation (USA; lines named NFxxx; [30]) or produced at the “Institut des Sciences du Végétal” (ISV, CNRS, Gif sur Yvette, France) and screened at the “Agroécologie” institute (INRA, Dijon, France; lines with other names than NFxxx). The seeds were scarified on sandpaper, sterilized for 20 min in bleach (12% [v/v] sodium hypochlorite), and thoroughly rinsed in sterile water. The seeds were then stratified at 4°C for one day and then germinated at 25°C in the dark on inverted water agar plates. The seedlings were grown *in vitro* in a growth chamber at 25°C with a 16 h light period and a 150 µE intensity on N-deprived media (“i”, [42]; or Fahraeus, [43]), on an N-rich medium (Fahraeus with NH_4_NO_3_ 10 mM), or on a N- and C-rich medium (“Lateral-Root-Inducing Medium”, LRIM; [42]) depending on the experiment. Alternatively, the plants were grown in a greenhouse (25°C, 16 h 150 µE light period, 60%–70% humidity) in soil or in pots containing a perlite∶sand mixture (3∶1) and watered every two days with “i” or “SN/2” media [42], depending on the experiment. The nodulation experiments used the *Sinorhizobium meliloti* 1021 strain (OD_600nm_ = 0.05) as described in [42].

### Transposon display, genotyping, RT-PCR and cloning

Plant genomic DNA was extracted from the leaves as described by [44], and Flanking Sequence Tags (FSTs) that were linked to *Tnt1* insertions were identified using the transposon display PCR method as described in [45] using the EcoR1 or Ase1 restriction enzymes. The transposon display, genotyping PCRs, and the sequencing of the different mutant alleles were performed using the primers that are listed in S1 Table. The RT-PCRs and real-time RT-PCRs were performed as described in [42] with the primers that are indicated in S1 Table.

To generate the Mt*CRA2* reporter transcriptional fusion, an 1800-bp sequence upstream of the CRA2 start codon was identified, corresponding to the upstream region of the Medtr3g110840.1 open reading frame (*M. truncatula* genome Mt4.0v1, http://www.jcvi.org/medicago/index.php). This region was amplified by PCR using a high-fidelity polymerase (Phusion, Thermo Scientific) and primers pCRA2-F and pCRA2-R (S1 Table). The promoter was cloned using Gateway technology initially into the pTOPO-Entry vector (Invitrogen) and then into the pkGWFS7 vector (https://gateway.psb.ugent.be/search) carrying a Green Fluorescent Protein (GFP) - β Glucuronidase (GUS) fusion downstream of the cloning recombination site.

### Grafting, root apex excision, and composite plants

The graftings were performed as described in the “cuttings and grafts” chapter of the *Medicago* handbook (http://www.noble.org/medicagohandbook/). The grafts, which were initially generated *in vitro*, were transferred after three weeks to pots containing a perlite∶sand mixture (3∶1) that was imbibed with the “i” solution in a growth chamber (same conditions as described above). After three weeks, the root length and root dry weight (60°C for 48 h) were measured.

For root apex excision, the roots were sectioned at one centimeter from the root tips one or three days post germination and placed in the “i” medium in a growth chamber (same conditions as described above). The number of lateral roots was quantified one to seven days post root tip excision using the ImageJ software (http://imagej.nih.gov/ij/).

To generate composite plants, constructs were introduced into the *Agrobacterium rhizogenes* ARqua1 strain and used for *M. truncatula* root transformation as described in [46]. Transgenic roots were selected on kanamycin (25 mg/L) for two weeks, and composite plants were then transferred onto growth papers (Mega International, http://www.mega-international.com/) on “i” medium for four to six days and, depending on the experiment, were inoculated by *S. meliloti* as described in [42].

### Microscopy analyses, histological stainings and *in situ* hybridization

The roots or stems were cut into 5-mm segments and immediately embedded in 3% agarose (Euromedex). A vibratome (VT1200S, Leica) was used to generate 100 µm-thick cross-sections. Toluidine blue and aniline blue (Sigma) stainings were performed by incubating sections for 3 to 5 min in 1% toluidine blue or in 0.005% aniline blue and subsequently washing the sections twice with distilled water. The sections were viewed under bright-field illumination or under UV excitation (365 nm), respectively, for toluidine blue or for aniline blue stainings using a Leica DMI 6000B inverted microscope. The phloroglucinol–HCl reagent was prepared by mixing 2 volumes of 2% (w/v) phloroglucinol (Sigma) in 95% ethanol with one volume of concentrated HCl and observation under bright-field illumination (DMI 6000B, Leica). Callose fluorescence was visualized using a 405 nm excitation, and emission was collected between 480 and 515 nm (DMI 6000B, Leica). The amyloplasts were detected using Lugol (Sigma) staining and visualization under bright field illumination.

To examine the detailed cellular organization of *M. truncatula* root apices, the roots were stained using a modified Pseudo Schiff-Propidium Iodide (PS-PI) staining protocol as described by [47]. Longitudinal optical sections were obtained using a Leica TCS SP2 confocal laser-scanning microscope using a 488 nm excitation, and emission was collected between 520 and 720 nm. The root meristem size was estimated as the number of cells in the outer cortex from the location of the tip to the first elongating cell using the ImageJ software. The length of the elongated cortical cells in the mature root region was also measured.

The GUS activity was revealed as described by (48] and observed under bright-field illumination (DMI 6000B, Leica). In addition, using the AOBS reflection mode of a Leica TCS SP8 spectral confocal laser-scanning microscope, the GUS precipitate reflectance was analyzed as described in [47] with a 488 nm excitation and a collection of the reflection signal between 485 and 491 nm on PS-PI counterstained samples (detected as described above).

*In situ* hybridization was performed as described in [48] with the probe as indicated in S1 Table.

### Nitrogen fixation activity

To measure nitrogenase activity of symbiotic nodules, an Acetylene Reduction Assay (ARA) was performed on individual plants with a protocol that was derived from [49]. One month after inoculation with *Rhizobium*, the plants were placed into 10-ml glass vials that were sealed with rubber septa. Acetylene was injected into each vial, and after 1 h of incubation at room temperature, the produced ethylene was measured using Gas Chromatography (7820A, Agilent technologies).

### Phylogenetic and statistical analyses

Phylogenetic analyses were performed using the SeaView package (v.4.4.0; [50]). The full-length protein sequences that were retrieved from the NCBI database were aligned using Muscle and optimized with the Gblocks software. Phylogenetic relationships were defined using a maximum likelihood approach. The tree was built with the PhyML software using the LG substitution model and four substitution rate categories. Support for each node was gained by approximate likelihood ratio tests (aLRT SH-like; [51]). The XP_001698687.1 RLK from *Chlamydomonas patens*, which was identified by a BLAST search of NCBI (E-value  =  1e-40), was used as an outgroup to anchor the tree.

The statistical analyses were performed with non-parametric tests (Mann-Whitney when n = 2 independent samples and Kruskal and Wallis when n>2 independent samples).

## Supporting Information

S1 Figure Allelism tests and root system architecture phenotypes of the various *cra2* mutant alleles. **A.** Representative examples of wild-type (WT) and *cra2* (alleles 1 to 10) root systems that were grown *in vitro* for two weeks on an N-deprived “i” medium [42]. The blue arrowheads are alleles that are tagged by the *Tnt1* retro-element insertion; the green arrowheads are alleles that are tagged by another insertional element; and the yellow arrowhead is an allele containing a deletion of one nucleotide. Bars  = 0,5 cm. **B.** Allelism test between different *cra2* alleles as well as with the previously described *cra1* mutant [32]. “yes” means that both of the mutants are allelic.
(PDF)

S2 Figure Detail of the cone-shaped transition zone in *Medicago truncatula* root apical meristems. Detail of the wild-type (WT) apical meristem transition zone (**B**) of the root that is shown in Fig. 2A (**A**). The roots were stained with Propidium Iodide to visualize the cell walls. The arrowhead indicates the apical position of the “cone-shaped” transition zone between the cell proliferation zone (CPZ) and the cell elongation zone (CEZ). Bars  = 100 µm.
(PDF)

S3 Figure Expression of the root apical meristem markers. **A.** Amyloplast accumulation in Wild-Type (WT) and *cra2-1* root apical meristems revealed by Lugol staining. **B.** Expression of the P*_WOX5_*:GUS transcriptional fusion in the WT and *cra2-1* root apical meristems. **C.** Real-time RT-PCR analysis of *WOX5* expression in the WT and *cra2-1* or *cra2-2* roots. *ACTIN11*, *RBP1* and *H3L* genes were used as references [48]. The expression was normalized relative to that of the WT, and the error bars represent standard deviations (n = 3).
(PDF)

S4 Figure *cra2* nodules are elongated and fix nitrogen. **A.** Picture of a representative elongated nodule from a Wild-Type (WT) or a *cra2-1* plant. Bar  = 500 µm. **B.** Nitrogen fixation activity of the WT and *cra2* plants (*cra2-1* and *cra2-2* alleles) six weeks post-inoculation with Rhizobium was determined using an Acetylene Reduction Assay (ARA). **C.** Specific nitrogen-fixation activity of WT and *cra2* nodules (*cra2-1* and *cra2-2* alleles) from plants shown in (**B**), corresponding to the ARA activity per milligram of nodule. In **B** and **C**, a Kruskal and Wallis test was performed (α<5%; n = 10), and the letters indicate significant differences.
(PDF)

S5 Figure *CRA2* gene structure and mutant allele location. **A**, Predicted *CRA2* gene model (FGenesh) and localization of the 10 mutant alleles that were identified by forward or reverse genetic screens. The blue arrowheads are alleles that are tagged by the *Tnt1* retro-element insertion; the green arrowheads are alleles that are tagged by another insertional element; and the yellow arrowhead is an allele containing a deletion of one nucleotide. Bar  = 250 nucleotides; TSS =  predicted Transcription Start Site; polyA: predicted polyadenylation site. **B**, Nucleotide sequence of the *CRA2* genomic region (from the predicted initial ATG start codon to the stop codon) locating the 10 mutant alleles (arrowheads; position related to the predicted ATG). **C**, Prediction (FGenesh) of a splicing site variant mutation in the *cra2-2* allele carrying a single-nucleotide deletion. Red box (1): WT Exon 1; Grey box (2): new exon that was predicted from the new splicing site. The arrows represent the primers that were used for the RT-PCR as shown in (**D**). **D**, RT-PCR analysis of the region containing the predicted splicing site in the *cra2-2* allele. No differential splicing was detected including after sequencing of the PCR product. **E**, Sequence of the CRA2 protein. The arrowhead shows the truncated protein that was generated by a frameshift in the *cra2-2* allele carrying a single-nucleotide deletion.
(PDF)

S6 Figure *CRA2* expression in various plant organs and growth conditions. The Mtr.38398.1.S1_at probe corresponding to the *CRA2* gene on the *M. truncatula* Affymetrix arrays is shown for the selected organs (including both above- and below-ground organs) and experimental conditions that are available from the MtGEA (*Medicago truncatula* Gene Expression Atlas database).
(PDF)

S7 Figure *cra2* roots and shoots do not present any detectable defect in vascular bundle patterning. **A**–**G**, Representative examples of stem (**A–G**) or root (**D–F**) transversal sections of wild-type (WT) and *cra2-1* plants that were grown for two months and observed after different stainings: **A** and **D**, phloroglucinol staining lignin in red and sclerenchyma in white; **B** and **E**, aniline blue staining callose in blue under UV illumination; and **C**, **F** and **G**, toluidine blue staining xylem (Xy) and phloem (Phl) in blue and sclerenchyma (scl) in violet (the detail of a stem vascular bundle is shown in **G**). Bars  = 150 µm in **A** and **B**; 50 µm in **C–G**. **H**, Quantification of the diameter of the roots and root steles based on transversal sections at one cm above the root apex in the WT and *cra2-1* plants that were grown in a greenhouse on a perlite-sand mixture for one month. The error bars represent standard deviations, and a Kruskal and Wallis test was used to determine the significant differences (indicated by letters, α<5%, n = 15).
(PDF)

S1 Table Primers list. *In italics: T7 promoter.*
(PDF)
