# Supplementary material for: Local and Systemic Regulation of Plant Root System Architecture and Symbiotic Nodulation by a Receptor-Like Kinase
Source: PLoS Genet. 2014 Dec 18;10(12):e1004891. doi: 10.1371/journal.pgen.1004891 (PMC4270686; doi:10.1371/journal.pgen.1004891)
Supplement: S1 Figure — Allelism tests and root system architecture phenotypes of the various cra2 mutant alleles. A. Representative examples of wild-type (WT) and cra2 (alleles 1 to 10) root systems that were grown in vitro for two weeks on an N-deprived “i” medium [42]. The blue arrowheads are alleles that are tagged by the Tnt1 retro-element insertion; the green arrowheads are alleles that are tagged by another insertional element; and the yellow arrowhead is an allele containing a deletion of one nucleotide. Bars = 0,5 cm. B. Allelism test between different cra2 alleles as well as with the previously described cra1 mutant [32]. “yes” means that both of the mutants are allelic. (PDF) [file pgen.1004891.s001.pdf]

A

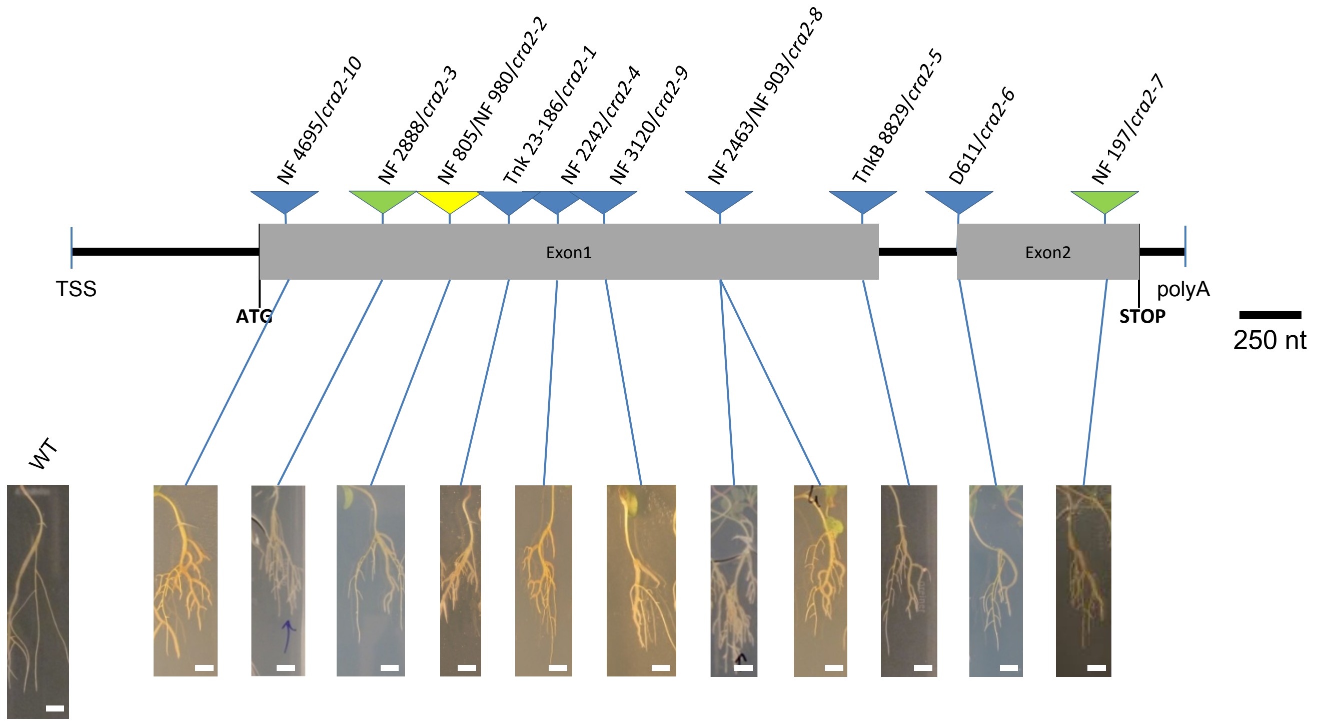

B

	cra2.2	*cra2.5*	*cra2.6*	*cra1.1*
cra2.1	yes	yes	yes	no
cra2.2		yes	yes	no
cra2.5			yes	no
cra2.6				no

Supplementary Figure 1. Allelism tests and root system architecture phenotypes of the various *cra2* mutant alleles.
A. Representative examples of wild-type (WT) and *cra2* (alleles 1 to 10) root systems that were grown *in vitro* for two weeks on an N-deprived "i" medium (42). The blue arrowheads are alleles that are tagged by the *Tnt1* retro-element insertion; the green arrowheads are alleles that are tagged by another insertional element; and the yellow arrowhead is an allele containing a deletion of one nucleotide. Bars = 0,5 cm.
B. Allelism test between different *cra2* alleles as well as with the previously described *cra1* mutant (32). "yes" means that both of the mutants are allelic.
