# Supplementary material for: Local and Systemic Regulation of Plant Root System Architecture and Symbiotic Nodulation by a Receptor-Like Kinase
Source: PLoS Genet. 2014 Dec 18;10(12):e1004891. doi: 10.1371/journal.pgen.1004891 (PMC4270686; doi:10.1371/journal.pgen.1004891)
Supplement: S2 Figure — Detail of the cone-shaped transition zone in Medicago truncatula root apical meristems. Detail of the wild-type (WT) apical meristem transition zone (B) of the root that is shown in Fig. 2A (A). The roots were stained with Propidium Iodide to visualize the cell walls. The arrowhead indicates the apical position of the “cone-shaped” transition zone between the cell proliferation zone (CPZ) and the cell elongation zone (CEZ). Bars = 100 µm. (PDF) [file pgen.1004891.s002.pdf]

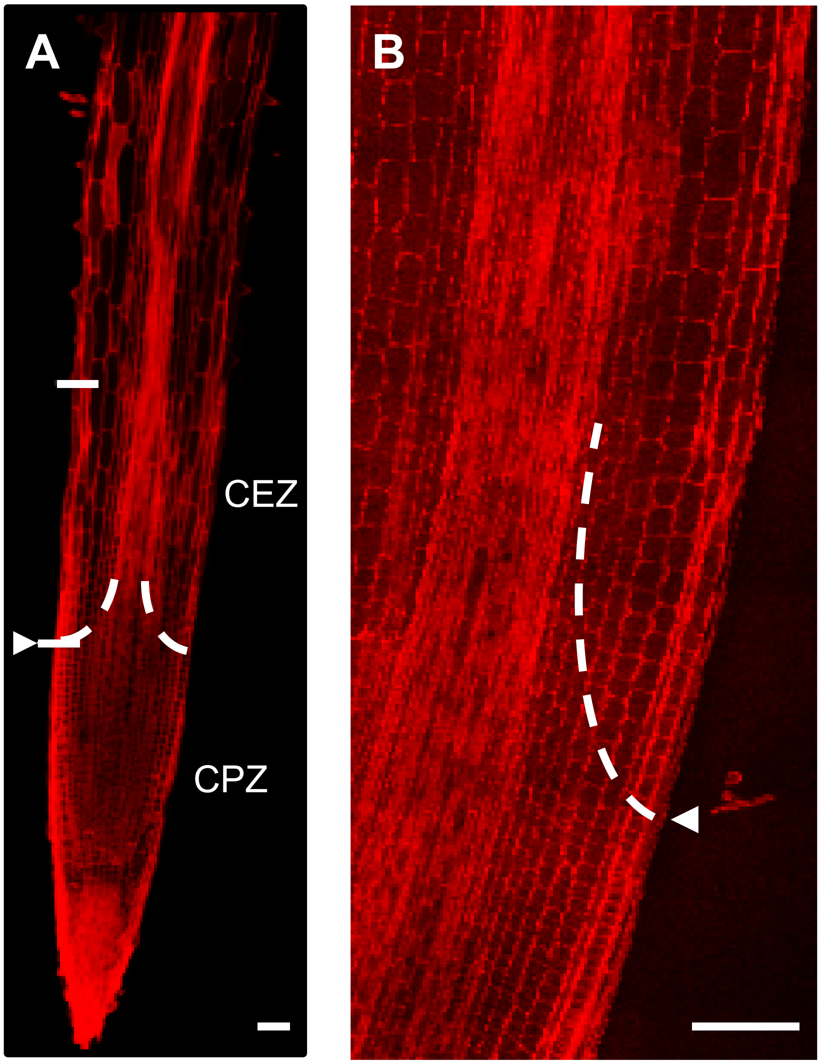

Supplementary Figure 2. Detail of the cone-shaped transition zone in *Medicago truncatula* root apical meristems

Detail of the wild-type (WT) apical meristem transition zone (**B**) of the root that is shown in Figure 2A (**A**). The roots were stained with Propidium Iodide to visualize the cell walls. The arrowhead indicates the apical position of the "cone-shaped" transition zone between the cell proliferation zone (CPZ) and the cell elongation zone (CEZ). Bars = 100 µm.
