# Supplementary material for: Local and Systemic Regulation of Plant Root System Architecture and Symbiotic Nodulation by a Receptor-Like Kinase
Source: PLoS Genet. 2014 Dec 18;10(12):e1004891. doi: 10.1371/journal.pgen.1004891 (PMC4270686; doi:10.1371/journal.pgen.1004891)
Supplement: S3 Figure — Expression of the root apical meristem markers. A. Amyloplast accumulation in Wild-Type (WT) and cra2-1 root apical meristems revealed by Lugol staining. B. Expression of the PWOX5:GUS transcriptional fusion in the WT and cra2-1 root apical meristems. C. Real-time RT-PCR analysis of WOX5 expression in the WT and cra2-1 or cra2-2 roots. ACTIN11, RBP1 and H3L genes were used as references [48]. The expression was normalized relative to that of the WT, and the error bars represent standard deviations (n = 3). (PDF) [file pgen.1004891.s003.pdf]

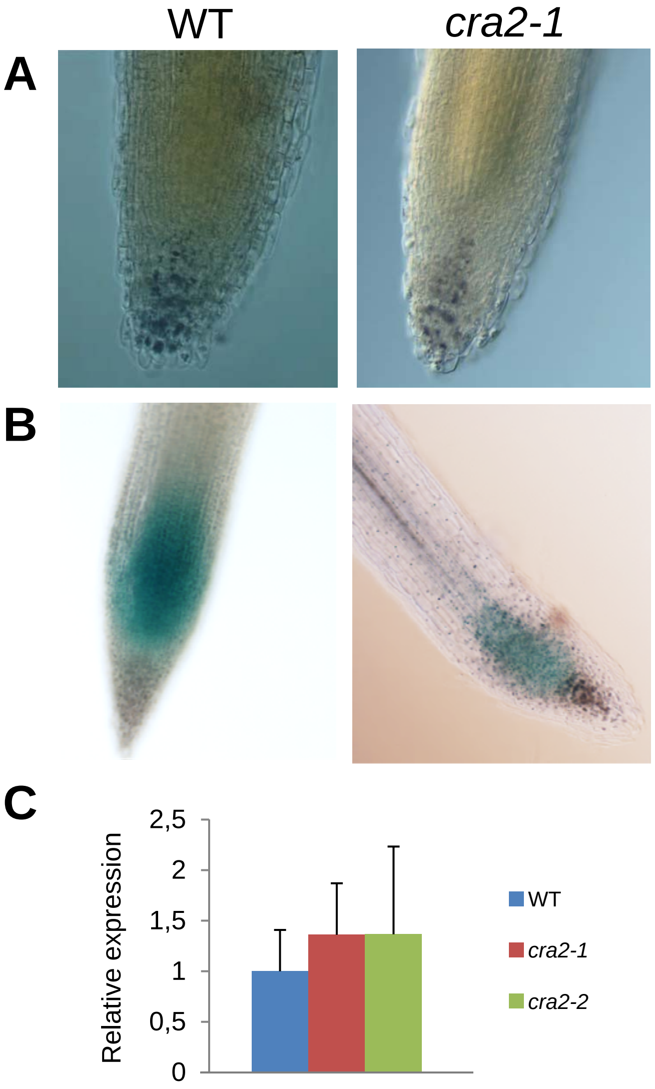

Supplementary Figure 3. Expression of the root apical meristem markers
A. Amyloplast accumulation in Wild-Type (WT) and *cra2-1* root apical meristems revealed by Lugol staining.
B. Expression of the P_{WOX5}:GUS transcriptional fusion in the WT and *cra2-1* root apical meristems.
C. Real-time RT-PCR analysis of *WOX5* expression in the WT and *cra2-1* or *cra2-2* roots. *ACTIN11*, *RBP1* and *H3L* genes were used as references (48). The expression was normalized relative to that of the WT, and the error bars represent standard deviations (n = 3).
