# Supplementary material for: Local and Systemic Regulation of Plant Root System Architecture and Symbiotic Nodulation by a Receptor-Like Kinase
Source: PLoS Genet. 2014 Dec 18;10(12):e1004891. doi: 10.1371/journal.pgen.1004891 (PMC4270686; doi:10.1371/journal.pgen.1004891)
Supplement: S4 Figure — cra2 nodules are elongated and fix nitrogen. A. Picture of a representative elongated nodule from a Wild-Type (WT) or a cra2-1 plant. Bar = 500 µm. B. Nitrogen fixation activity of the WT and cra2 plants (cra2-1 and cra2-2 alleles) six weeks post-inoculation with Rhizobium was determined using an Acetylene Reduction Assay (ARA). C. Specific nitrogen-fixation activity of WT and cra2 nodules (cra2-1 and cra2-2 alleles) from plants shown in (B), corresponding to the ARA activity per milligram of nodule. In B and C, a Kruskal and Wallis test was performed (α<5%; n = 10), and the letters indicate significant differences. (PDF) [file pgen.1004891.s004.pdf]

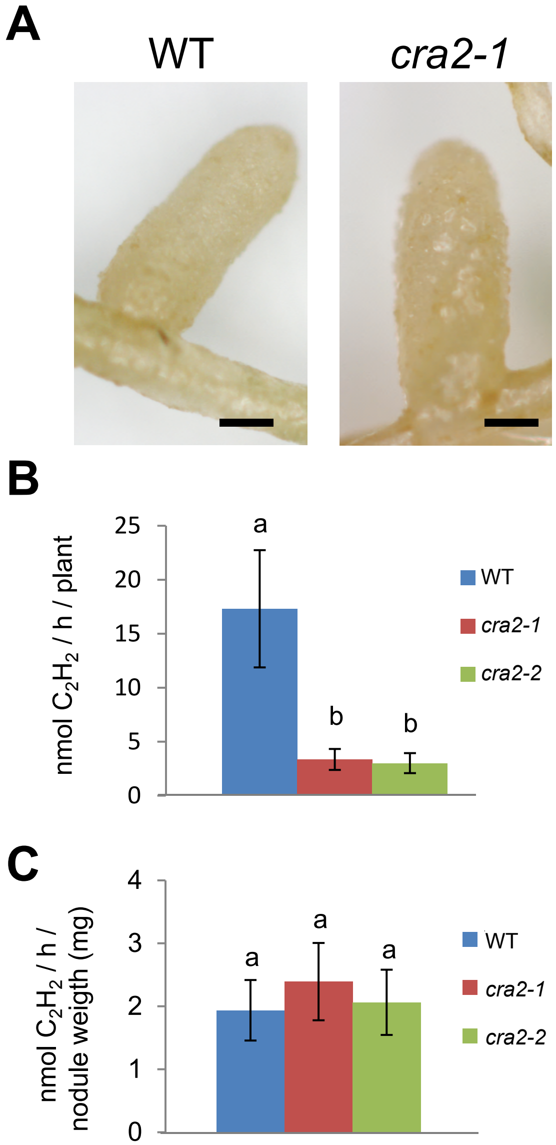

Supplementary Figure 4. ***cra2*** **nodules are elongated and fix nitrogen**
A. Picture of a representative elongated nodule from a Wild-Type (WT) or a *cra2-1* plant. Bar = 500 µm
B. Nitrogen fixation activity of the WT and *cra2* plants (*cra2-1* and *cra2-2* alleles) six weeks post-inoculation with Rhizobium was determined using an Acetylene Reduction Assay (ARA).
C. Specific nitrogen-fixation activity of WT and *cra2* nodules (*cra2-1* and *cra2-2* alleles) from plants shown in (**B**), corresponding to the ARA activity per milligram of nodule.
In **B** and **C**, a Kruskal and Wallis test was performed ($\alpha < 5$ %; n = 10), and the letters indicate significant differences.
