# Supplementary material for: Local and Systemic Regulation of Plant Root System Architecture and Symbiotic Nodulation by a Receptor-Like Kinase
Source: PLoS Genet. 2014 Dec 18;10(12):e1004891. doi: 10.1371/journal.pgen.1004891 (PMC4270686; doi:10.1371/journal.pgen.1004891)
Supplement: S5 Figure — CRA2 gene structure and mutant allele location. A, Predicted CRA2 gene model (FGenesh) and localization of the 10 mutant alleles that were identified by forward or reverse genetic screens. The blue arrowheads are alleles that are tagged by the Tnt1 retro-element insertion; the green arrowheads are alleles that are tagged by another insertional element; and the yellow arrowhead is an allele containing a deletion of one nucleotide. Bar = 250 nucleotides; TSS = predicted Transcription Start Site; polyA: predicted polyadenylation site. B, Nucleotide sequence of the CRA2 genomic region (from the predicted initial ATG start codon to the stop codon) locating the 10 mutant alleles (arrowheads; position related to the predicted ATG). C, Prediction (FGenesh) of a splicing site variant mutation in the cra2-2 allele carrying a single-nucleotide deletion. Red box (1): WT Exon 1; Grey box (2): new exon that was predicted from the new splicing site. The arrows represent the primers that were used for the RT-PCR as shown in (D). D, RT-PCR analysis of the region containing the predicted splicing site in the cra2-2 allele. No differential splicing was detected including after sequencing of the PCR product. E, Sequence of the CRA2 protein. The arrowhead shows the truncated protein that was generated by a frameshift in the cra2-2 allele carrying a single-nucleotide deletion. (PDF) [file pgen.1004891.s005.pdf]

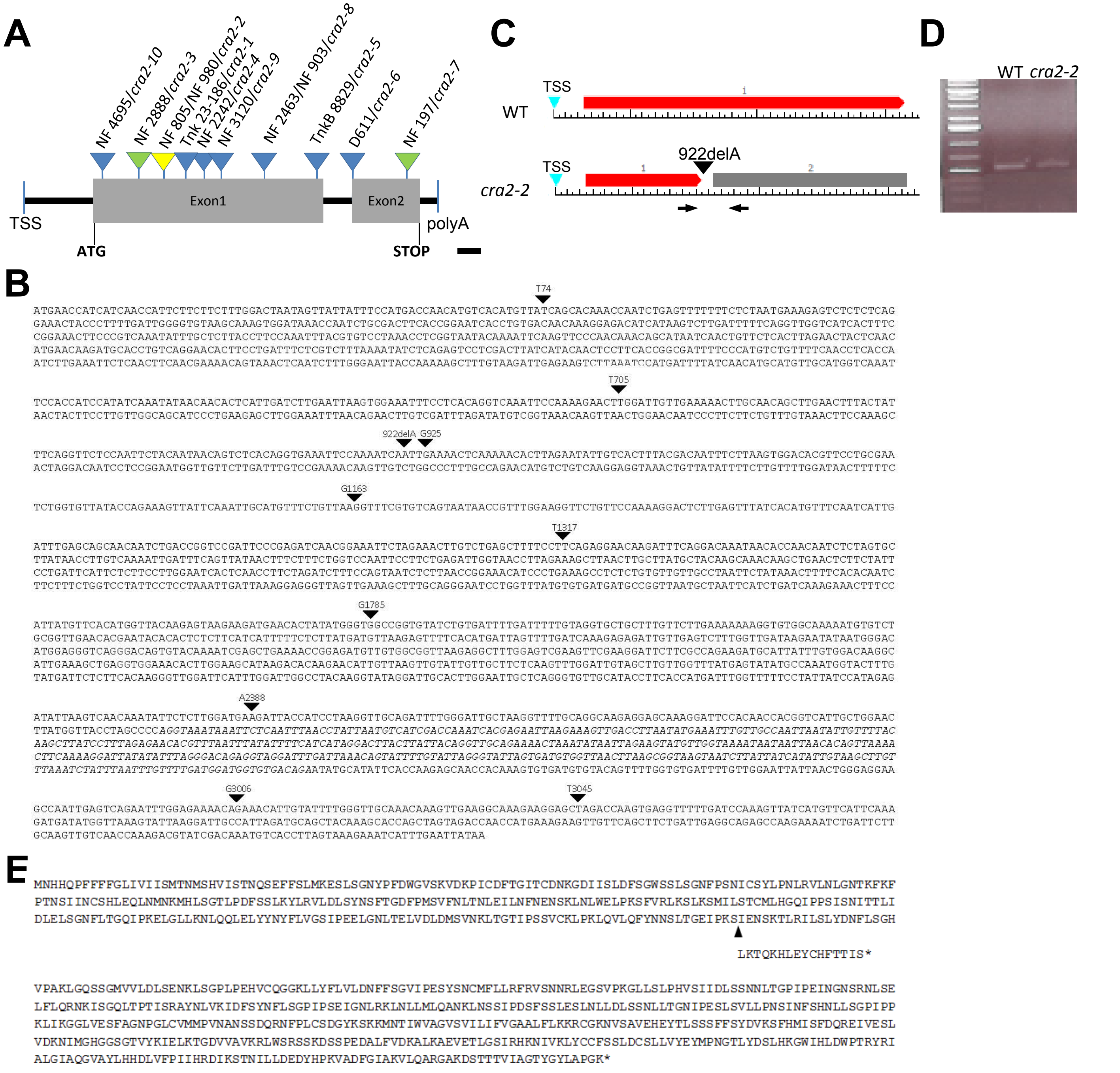

Supplementary Figure 5. *CRA2* gene structure and mutant allele location

A, Predicted *CRA2* gene model (FGenesh) and localization of the 10 mutant alleles that were identified by forward or reverse genetic screens. The blue arrowheads are alleles that are tagged by the *Tnt1* retro-element insertion; the green arrowheads are alleles that are tagged by another insertional element; and the yellow arrowhead is an allele containing a deletion of one nucleotide. Bar = 250 nucleotides; TSS= predicted Transcription Start Site; polyA: predicted polyadenylation site.

B, Nucleotide sequence of the *CRA2* genomic region (from the predicted initial ATG start codon to the stop codon) locating the 10 mutant alleles (arrowheads; position related to the predicted ATG).

C, Prediction (FGenesh) of a splicing site variant mutation in the *cra2-2* allele carrying a single-nucleotide deletion. Red box (1): WT Exon 1; Grey box (2): new exon that was predicted from the new splicing site. The arrows represent the primers that were used for the RT-PCR as shown in (**D**).

D, RT-PCR analysis of the region containing the predicted splicing site in the *cra2-2* allele. No differential splicing was detected including after sequncing of the PCR product.

E, Sequence of the CRA2 protein. The arrowhead shows the truncated protein that was generated by a frameshift in the *cra2-2* allele carrying a single-nucleotide deletion.
