# Supplementary material for: Local and Systemic Regulation of Plant Root System Architecture and Symbiotic Nodulation by a Receptor-Like Kinase
Source: PLoS Genet. 2014 Dec 18;10(12):e1004891. doi: 10.1371/journal.pgen.1004891 (PMC4270686; doi:10.1371/journal.pgen.1004891)
Supplement: S7 Figure — cra2 roots and shoots do not present any detectable defect in vascular bundle patterning. A–G, Representative examples of stem (A–G) or root (D–F) transversal sections of wild-type (WT) and cra2-1 plants that were grown for two months and observed after different stainings: A and D, phloroglucinol staining lignin in red and sclerenchyma in white; B and E, aniline blue staining callose in blue under UV illumination; and C, F and G, toluidine blue staining xylem (Xy) and phloem (Phl) in blue and sclerenchyma (scl) in violet (the detail of a stem vascular bundle is shown in G). Bars = 150 µm in A and B; 50 µm in C–G. H, Quantification of the diameter of the roots and root steles based on transversal sections at one cm above the root apex in the WT and cra2-1 plants that were grown in a greenhouse on a perlite-sand mixture for one month. The error bars represent standard deviations, and a Kruskal and Wallis test was used to determine the significant differences (indicated by letters, α<5%, n = 15). (PDF) [file pgen.1004891.s007.pdf]

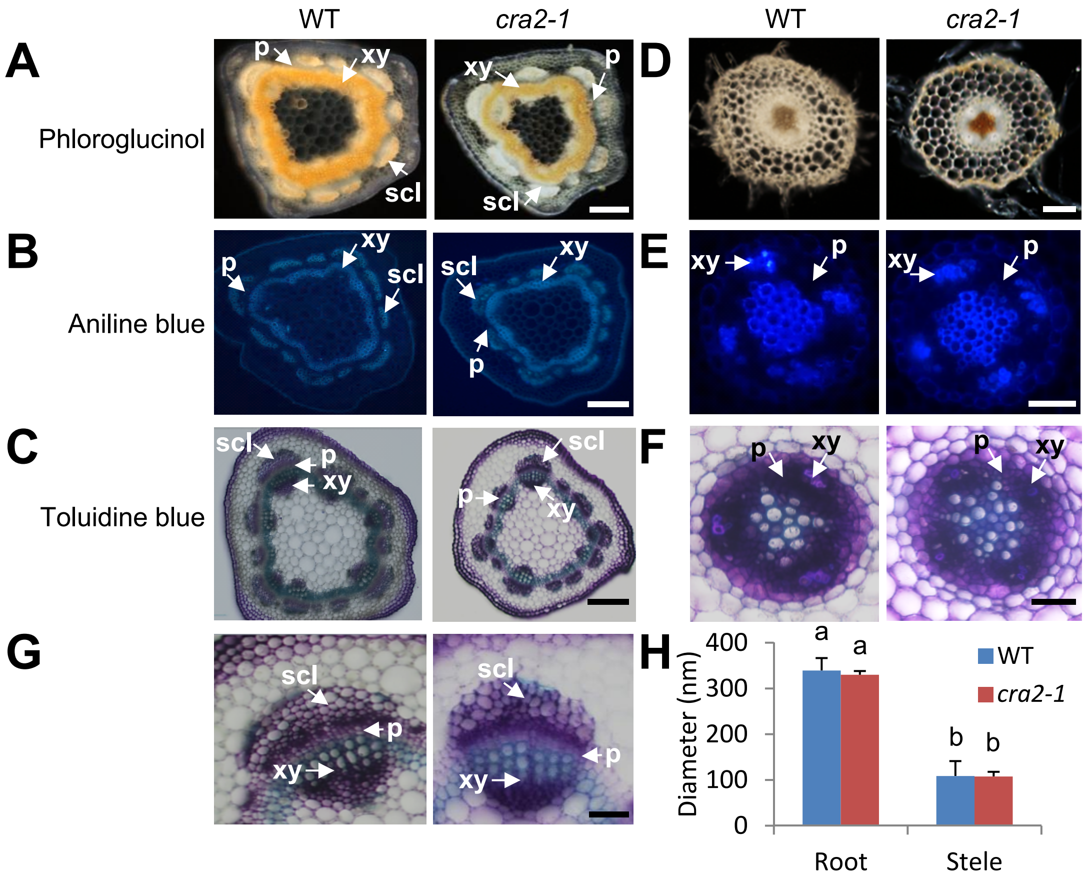

Supplementary Figure 7. ***cra2*** **roots and shoots do not present any detectable defect in vascular bundle patterning**

A-G, Representative examples of stem (**A-G**) or root (**D-F**) transversal sections of wild-type (WT) and *cra2-1* plants that were grown for two months and observed after different stainings: **A** and **D**, phloroglucinol staining lignin in red and sclerenchyma in white; **B** and **E**, aniline blue staining callose in blue under UV illumination; and **C**, **F** and **G**, toluidine blue staining xylem (Xy) and phloem (Phl) in blue and sclerenchyma (scl) in violet (the detail of a stem vascular bundle is shown in **G**). Bars = 150 µm in **A** and **B**; 50 µm in **C-G**.

H, Quantification of the diameter of the roots and root steles based on transversal sections at one cm above the root apex in the WT and *cra2-1* plants that were grown in a greenhouse on a perlite-sand mixture for one month. The error bars represent standard deviations, and a Kruskal and Wallis test was used to determine the significant differences (indicated by letters, $\alpha < 5\%$, n = 15).
