# Supplementary material for: Local and Systemic Regulation of Plant Root System Architecture and Symbiotic Nodulation by a Receptor-Like Kinase
Source: PLoS Genet. 2014 Dec 18;10(12):e1004891. doi: 10.1371/journal.pgen.1004891 (PMC4270686; doi:10.1371/journal.pgen.1004891)
Supplement: S6 Figure — CRA2 expression in various plant organs and growth conditions. The Mtr.38398.1.S1_at probe corresponding to the CRA2 gene on the M. truncatula Affymetrix arrays is shown for the selected organs (including both above- and below-ground organs) and experimental conditions that are available from the MtGEA (Medicago truncatula Gene Expression Atlas database). (PDF) [file pgen.1004891.s006.pdf]

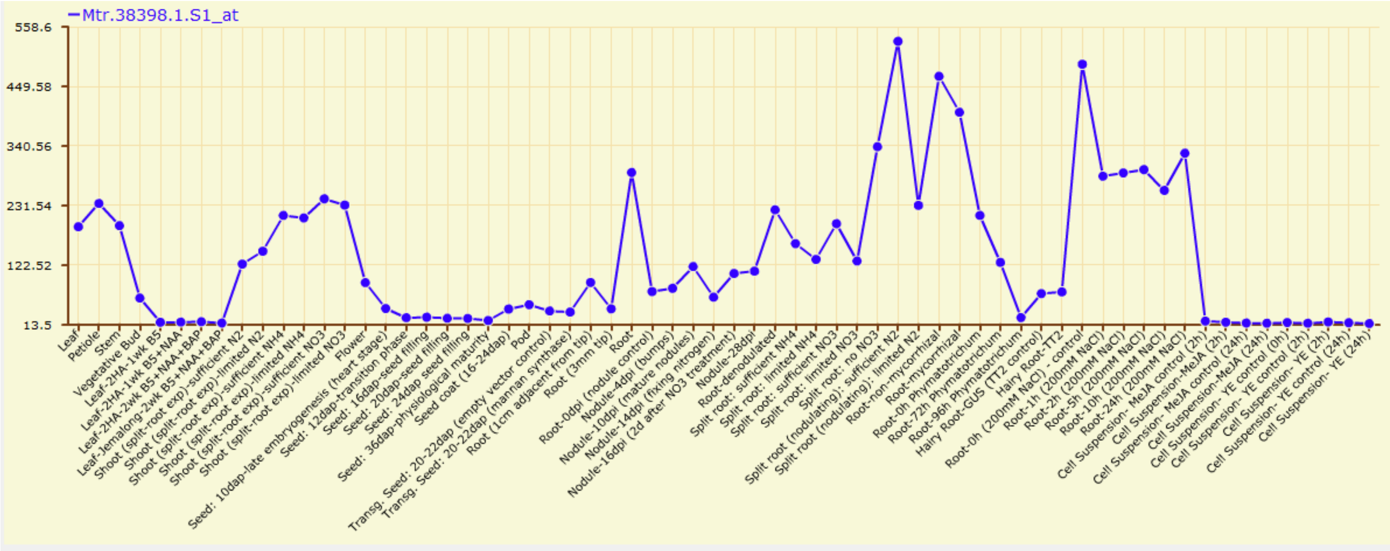

Supplementary Figure 6. ***CRA2*** **expression in various plant organs and growth conditions**

The Mtr.38398.1.S1_at probe corresponding to the *CRA2* gene on the *M. truncatula* Affymetrix arrays is shown for the selected organs (including both above- and below-ground organs) and experimental conditions that are available from the MtGEA (*Medicago truncatula* Gene Expression Atlas database).
