# Supplementary material for: Local and Systemic Regulation of Plant Root System Architecture and Symbiotic Nodulation by a Receptor-Like Kinase
Source: PLoS Genet. 2014 Dec 18;10(12):e1004891. doi: 10.1371/journal.pgen.1004891 (PMC4270686; doi:10.1371/journal.pgen.1004891)
Supplement: S1 Table — Primers list. In italics: T7 promoter. (PDF) [file pgen.1004891.s008.pdf]

Genotyping and sequencing

CRA2 1F	TTCAGATTTCCTTCCTCGGC
CRA2 1R	CATCTTTGCTAAAGAGGCTTTATGT
CRA2 2F	AAATGTGGGAGCAGCAATTC
CRA2 2R	TGGAAAGGTTTGAGTGGATG
CRA2 3F	TTCCTCTTTACACATAGTGG
CRA2 3R	CGGAAAGTGATGACCAACCT
CRA2 4F	GGTCAAATTCCACCATCCAT
CRA2 4R	CCAGAGAAAAAGTTATCCAAAACAA
CRA2 5F	GGAGACATCATAAGTCTTG
CRA2 5R	TGCAAGTTTTTCAACAATCCA
CRA2 6F	GGTCAAATTCCAAAAGAACTTGG
CRA2 6R	GGAACAGAACCTTCCAAACG
CRA2 7F	ACTTCCTTGTTGGCAGCATC
CRA2 7R	CCTTTTGGAACAGAACCTTCC
CRA2 8F	GGTTTCGTGTCAGTAATAACCG
CRA2 8R	TCCCTGCAAAGCTTTCAACT
CRA2 9F	TCCTAAATTGATTAAAGGAGG
CRA2 9R	CCAAGTGTTTCCACCTCAGC
CRA2 10F	AAGATGCATTATTTGTGGACAAGGC
CRA2 10R	TTGGCAACAAATTTCATATTAAGG
CRA2 11F	TGTCATCGACCAAATCACG
CRA2 11R	AGCTCCTTCTTTGCCTTCAA
CRA2 12F	TTGATGGATGGTGTGACAGAA
CRA2 12R	GCAGTCTCTCTTTTGTTGTGATG
CRA2 13F	TGCTCCCTCTTACCCCTTAAT
CRA2 13R	AGGGACAGAGGTAGGATTTGA
CRA2 14F	TTGAAGGCAAAGAAGGAGCT
CRA2 14R	AACATTGTATTTTGGGTTGC
CRA2 15F	ATGTGTGATGATGCCGGTTA
CRA2 15R	AGGATTGCACTTGGAATTGC
CRA2 16F	GGATTCATTTGGATTGGCC
CRA2 16R	GGCTTCCTCCCAGTTAATAATTCC
CRA2 17F	GAACATGTCTGTCAAGGAGG
CRA2 17R	TCCCTGCAAAGCTTTCAACT
CRA2 18F	CGTTCCTGCGAAACTAGGAC
CRA2 18R	ACCGGCATCATCACACATAA
CRA2 19F	GGATTGTTGAAAAACTTGC
CRA2 19R	CCTCCTTGACAGACATGTTC
CRA2 20F	TTGTGAAAAATCGAAGAAAGGA
CRA2 20R	AATGCCACCAACAAAATGATC

Tnt1 display

LTR4	TACCGTATCTCGGTGCTACA
LTR6	GCTACCAACCAAACCAAGTCAA
LTR31	GCTCCTCTCGGGGTCGTGG
LTR51	CAAAGCTTCACCCTCTAAAGCC
Adaptator 1	CCCCTCGTAGACTGCGTACC
Eco-adaptor2	AATTGGTACGCAGTCTACG
Ase-adaptor2	TAGGTACGCAGTCTACGA
Eco-1	CTCGTAGACTGCGTACCAA
Eco-2	CGTAGACTGCGTACCAATT
Ase-1	CTCGTAGACTGCGTACCTA
Ase-2	CGTAGACTGCGTACCTAAT

CRA2 promoter cloning

pCRA2-F	CACCTTGGATTTGGATTTTTGTTGC
pCRA2-R	TGTGTTTGTTTTGTGGTTTGG

qRT-PCR

WOX5-5'	GCCTGATAGAGTGATTGAGAC
WOX5-3'	GGGTGTTCCATTTGTTCTCC

***in situ* hybridization**

CRA2 -F1	TCAGCACAAACCAATCTGAGT
CRA2 -T7-R1	*TGTAATACGACTCACTATAGGGC* TTGCTGTTTGTTGGGAACTTGA

In italics : T7 promoter
